# The repaired tetralogy of Fallot become adult: what should we expect

**DOI:** 10.1186/1749-8090-8-S1-O132

**Published:** 2013-09-11

**Authors:** E Angeli, FD Petridis, R Liberi, G Oppido, L Careddu, S Volpi, R Formigari, L Ragni, M Agulli, G Gargiulo

**Affiliations:** 1 Pediatric and Adult Congenital Cardiac Surgery Unit, S. Orsola-Malpighi Hospital, University of Bologna Medical School, Bologna, Italy; 2 Pediatric and Adult Congenital Cardiology Unit, S. Orsola-Malpighi Hospital, University of Bologna Medical School, Bologna, Italy; 3 Cardiosurgery Intensive Care Unit, S. Orsola-Malpighi Hospital, University of Bologna Medical School, Bologna, Italy

## Background

To define the evolution of repaired Tetralogy of Fallot (TOF) in adult patients.

## Methods

82 patients with repaired tetralogy of Fallot were collected from the database of our pediatric and congenital adult cardiology and cardiac surgery unit. Only patients older than 16 years of age at the time of the study were selected. All patients underwent complete surgical repair during childhood at a mean age of1.6±1.3 years. Forty-nine patients (71.9%) were treated with transannular patch, 17(23.2%) infundibular patch, 3(3.65%) endoventricular repair and 1(1.2%) with conduit between the right ventricle(RV) and the pulmonary artery(PA); 17/82(20.7%) of all patients required palliative BT shunt at birth before repair. Mean age at follow up was 23.7± 6.7 years. Follow-up schedule comprised clinical evaluation along with echocardiographic and cardiac-MR, quality of life and VO2 consumption assessment.

## Results

After the 16 years of age, 53/82pts (65%) didn’t require any surgical procedure, 29/82(35%) required reinterventions to reconstruct the right ventricle outflow tract. Associated residual VSD repair was performed in 1/29 pts, tricuspid valve repair in 2/29 and aortic valve repair in 1/29. Twenty-one patients (25.6%) required percutaneous procedures on pulmonary arteries, including pulmonary branch angioplasty in 11/21, pulmonary stent implantation in 8/21, percutaneous valve angioplasty in 2/21. All patients survived. None of patients developed ventricular failure. At cardiopulmonary exercise testing the peak VO2 was moderately impaired.

Mean follow up time was 7.8±6.6 years.

## Conclusion

Survival prospects for adults with repaired TOF in adult age are now excellent. Incidence of reinterventions is predominant on the right ventricle outflow tract, where timing and correct indications are mandatory to avoid heart failure development. Late functional health status is satisfactory and quality of life is nearly comparable with those of healthy patients.

